# Case Report: Telitacicept in treating a patient with NF155+ autoimmune nodopathy: a successful attempt to manage recurrent elevated sero-anti-NF155 antibodies

**DOI:** 10.3389/fimmu.2023.1279808

**Published:** 2023-10-30

**Authors:** Yijun Ren, Si Chen, Huan Yang

**Affiliations:** Department of Neurology, Xiangya Hospital, Central South University, Changsha, China

**Keywords:** autoimmune nodopathy, telitacicept, neurofascin-155, plasma cells, treatment

## Abstract

This report presents a case of a neurofascin-155 (NF155)+ autoimmune nodopathy (AN) patient who exhibited resistance to conventional treatments but responded positively to telitacicept therapy. Telitacicept, a dual inhibitor of B lymphocyte stimulator (BLyS) and A proliferation-inducing ligand (APRIL), suppressed the development and survival of plasma cells and mature B cells. The patient’s unique clinical features were consistent with NF155+ AN, showing limited response to standard treatments like rituximab and a recurrent significant increase in anti-NF155 antibody titers. Administering telitacicept (160mg, ih) led to an improvement in clinical symptoms, inflammatory neuropathy cause and treatment (INCAT) scale and inflammatory Rasch-built overall disability scale (I-RODS), and stabilized anti-NF155 antibody levels without a rebound. This case demonstrates telitacicept as a potential novel therapy for NF155+ AN, particularly when conventional treatments fail. Further investigation into its safety, efficacy, dosage, and treatment cycle in NF155+ AN is warranted.

## 1 Introduction

Autoimmune nodopathies (AN) were established as a novel diagnostic category in the 2021 European Academy of Neurology/Peripheral Nerve Society (EAN/PNS) guideline for chronic inflammatory demyelinating polyradiculoneuropathy (CIDP) (1). Characterized by antibodies against nodal-paranodal cell-adhesion molecules like Contactin-1 (CNTN1), contactin-associated protein 1 (Caspr1), neurofascin-140/186 (NF140/186), and neurofascin-155 (NF155) (2). NF is a key adhesion molecule between nodal and paranodal proteins, playing a crucial role in shaping the nodes of Ranvier (3). The pathologies mediated by these antibodies predominantly affect the nodal and paranodal regions of Ranvier and are typically devoid of inflammation and classical macrophage-mediated segmental demyelination (2, 4, 5). Clinically, AN differs from CIDP in terms of both its features and therapeutic approaches, leading to its classification as a distinct disease entity rather than a CIDP subgroup. The NF155+ nodopathy subtype of AN, primarily observed in young adults, exhibits unique symptoms such as ataxia and tremor (1, 2, 6). In the majority of cases, the predominant immunoglobulin (IgG) subclass of anti-NF155 antibodies is IgG4, which often leads to a lackluster response to intravenous immunoglobulin (IVIg) treatment (7).

Therapies like rituximab, plasma exchange (PE), and immunoadsorption (IA) have been effective in treating NF155+ AN patients (2, 8–10). However, recurrent symptoms and poor response in some cases emphasize the need for more targeted interventions. The treatment of NF155+ AN, distinct from traditional types of CIDP, remains a neuroimmunology challenge, often leading to limited outcomes and long-term dysfunction (5, 8, 10). Alternative strategies are required to enhance efficacy and quality of life.

Enhancing the prognosis of NF155+ AN requires more effective and long-lasting treatment options tailored to the patient population. Understanding immunomodulation and the node of Ranvier’s role enables the exploration of new therapies like telitacicept. Its multifaceted mechanisms include B-Cell regulation, suppressing B-cell survival and proliferation (11); Immune Response Modulation, balancing immune responses (12); and Chronic Inflammation Reduction, mitigating inflammation associated with NF155+ AN (13). These approaches may pave the way for personalized treatments for NF155+ AN patients.

In this context, we highlight a 14-year-old female NF155+ AN patient who showed a poor response to rituximab and PE but responded positively to telitacicept, illustrating the potential for this treatment in this particular patient group.

## 2 Case description

### 2.1 Clinical presentation and physical examination

A 14-year-old female patient with no medical history was admitted to our department with symptoms of progressive muscle weakness, distal numbness in both upper and lower limbs for three months, and dysphagia and slurred speech persisting for one month following a cold. Physical examination revealed cranial nerve involvement, distal weakness in all four limbs (grade 4 distally and grade 5- proximally), hypoesthesia, absent tendon reflexes, tremor, and ataxia. There was no evidence of muscle atrophy or fasciculation, and both pyramidal and meningeal irritation signs were negative. The patient’s modified Rankin Scale (mRS) score was 3 out of 5.

### 2.2 Clinical findings

**Figure 1:**
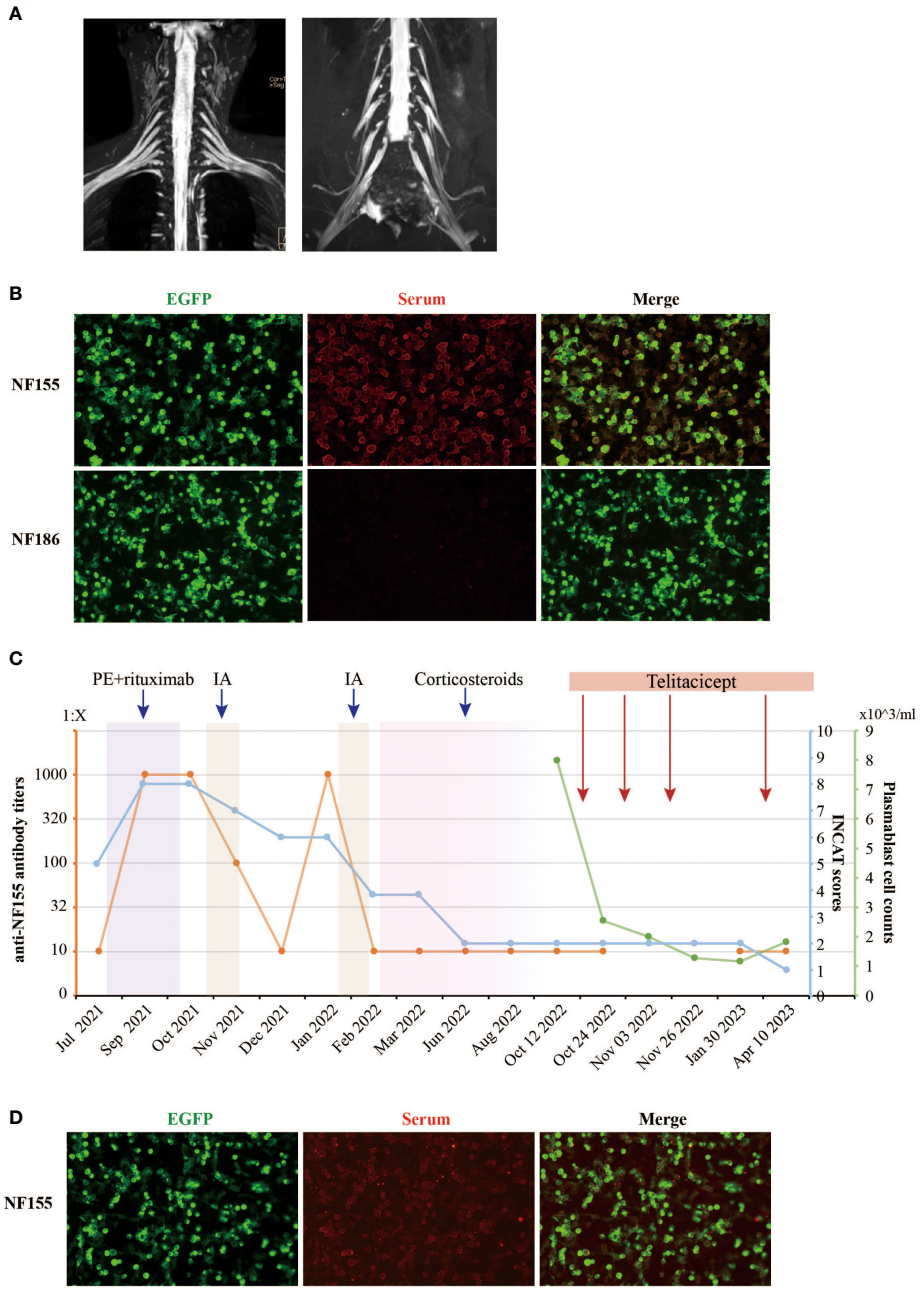
Clinical and laboratory parameters of the NF155+ AN patient treated with telitacicept. **(A)** The contrast-enhanced MRI images of the brachial and sacral plexuses nerve of the NF155+ AN patient. No thickening features of the nerve roots were observed. **(B)** The serum anti-NF155 antibodies and anti-NF186 antibodies were detected using the cell-based assay (CBA) in October 2021. The anti-NF155 was seropositive in the titer of 1:1000 (red), while the anti-NF186 was seronegative. EGFP is shown in green fluorescence. **(C)** The patient was diagnosed with NF155+ AN at the Department of Neurology, Xiangya Hospital, in July 2021. The right axis shows the level of anti-NF155 antibody titers in orange color. The first left axis shows the INCAT scores in blue color. The second left axis shows the peripheral plasmablast cell counts in green color. The rectangles in different colors, including purple, yellow, pink, and red, indicate different treatments the patient received during the period. **(D)** The serum anti-NF155 was seropositive in the titer of 1:10 (red) after telitacicept treatment in April 2023. PE, plasma exchange; IA, immunoadsorption; NF155, neurofascin-155; NF186, neurofascin-186; EGFP, Enhanced Green Fluorescent Protein.

An electromyogram (EMG) revealed significant peripheral nerve (PN) damage in limbs, with both motor and sensory involvement, axonal damage, and demyelination. Brachial plexus and sacral nerve magnetic resonance imaging (MRI) showed no thickening features of the nerve roots (**Figure 1A**). Cerebrospinal fluid (CSF) analysis displayed elevated protein levels at 1.82 g/L, while white blood cell count was normal. Serum tests were positive for IgG4 anti-NF155 antibodies (titer of 1:10) and IgG anti-GM4 antibodies, but negative for other neurofascin isoforms, contactins, and gangliosides. Tests for vascular endothelial growth factor (VEGF), free light chain protein, and M protein were normal, ruling out other diagnoses.

### 2.3 Diagnosis and initial treatment

Based on the patient’s clinical features and laboratory findings, a confident diagnosis of NF155+ AN was established, corroborated by strong evidence (1). The INCAT score was 5 at this time (3 for arm disabilities and 2 for leg disabilities).

The initial treatment regimen began with two sessions of PE over a three-day span, followed by the administration of mycophenolate mofetil (MMF) (0.5g bid). Regrettably, one month later, the patient’s symptoms, including weakness, continued to intensify. Serum anti-NF155 titers dramatically escalated from 1:10 to 1:1000+, accompanied by an increased INCAT score of 8 (4 for arms and 4 for legs). Complications also emerged, such as a cough and sputum, which were assessed as interstitial lung disease (ILD), a severe adverse reaction to MMF, necessitating the discontinuation of the medication.

The patient was readmitted for two more sessions of PE, followed by rituximab, which brought modest improvement (**Figure 1C**). However, anti-NF155 titers surprisingly surged to 1:1000++ after treatment (**Figure 1B**) and the INCAT score remained 8. Despite nine sessions of immunoadsorption (IA) reducing the titers and improving INCAT scores to 6, the titers rebounded again in January 2022, three months after IA therapy. After a second cycle of IA therapy, a total 9-month-long course of oral corticosteroids treatment was initiated at a dose of 30mg/day for 4 months, which showed great improvement in her clinical symptoms and a reduced INCAT score to 2. However, severe side effects occurred during the treatment, including acne and menstrual disturbances. The dose of oral corticosteroids was gradually reduced by 5mg/week until a dose of 5mg/day for 4 months before discontinuation. Overall, responses to treatments including PE, MMF, rituximab, IA and steroids remained suboptimal, and the case presented complex challenges.

At this stage, the clinical team was confronted with a daunting challenge: crafting a tailored and efficacious treatment approach to ameliorate her symptoms and manage the repeatedly elevated titers of serum anti-NF155, all while considering the unique needs and complexities of her case.

### 2.4 Therapeutic innovation

In seeking an explanation for the recurrent elevation of anti-NF155, the investigation of B cell subsets revealed significant findings. Following rituximab treatment, the CD19+ B cell counts were markedly reduced, while plasma blast cells (CD19+dimCD38+CD27+) constituted an abnormally high proportion of B cells at 77.3%. These plasma cells, serving as antibody-secreting cells (ASC), have the capacity to home to the bone marrow niche and transform into both short-lived plasmablasts and long-lived plasma cells, thus secreting antibodies over an extended duration (14). This led to the utilization of telitacicept, a novel therapeutic agent targeting BLyS and APRIL, known to inhibit the proliferation of B lymphocytes (11). A professor of neuroimmunologist evaluated the patient’s condition and responses to treatments. Treatment with telitacicept was initiated, consisting of four 160mg subcutaneous injections, all under the close monitoring of plasmablast cell counts (**Figure 1C**). Remarkably, the serum anti-NF155 titers remained stable at a low level of 1:10 during the telitacicept regimen (**Figure 1D**), with the patient’s symptoms showing gradual improvement and the INCAT scores declining to 1 (0 for arms and 1 for legs). The I-RODS also reflected this positive progress, with a score of 54 in a centile metric value.

## 3 Discussion

We present a case of an NF155+ AN patient who did not respond to commonly recommended treatments cited in existing literature but eventually responded to telitacicept. The clinical features of NF155+ AN patients significantly differ from typical CIDP patients, predominantly affecting young adults and manifesting in symptoms such as distal weakness, motor ataxia, tremor, high protein content in CSF, and limited or no response to IVIg treatment (1, 2, 8). Around a quarter of these patients suffered from cranial nerve involvement, including facial weakness and ophthalmoparesis (2). The patient we reported aligns with these clinical features, and the NF155+ AN diagnosis is unambiguous.

In NF155+ AN patients, IgG 4 anti-NF155 antibodies are predominant, leading to poor response to IVIg or steroids and favorable response to rituximab (15). In published studies, rituximab has led to decreased anti-NF155 antibody titers, and most patients have shown clinical improvement in INCAT and I-RODS scales. There is no definitive evidence regarding whether rituximab-treated anti-NF155 AN patients, pretreated with PE, benefit more (8). Some researchers believe that pretreatment with PE and IA may aid in antibody clearance and expedite recovery, although this requires further investigation. There are also reports of NF155+ AN patients producing anti-rituximab antibodies post-treatment, diminishing rituximab’s efficacy (16). In our case, the initial treatment with PE and rituximab was unsuccessful in improving the patient’s clinical symptoms, and anti-NF155 antibody titers even increased significantly (**Figure 1B**). This suggests that the treatment of NF155+ AN might be heterogeneous, and additional therapeutic strategies need to be explored.

According to previous studies, anti-NF155 antibodies are pathogenic (17), and their titers correlate with mRS, reflecting the neurological status of patients to some extent (8). Higher titers often signify more severe disease (18). With NF155-IgG4-seropositive patients prone to relapses that heavily affect walking ability, it is vital to reduce antibody titers and maintain them at low levels.

Telitacicept, a dual inhibitor, can bind to BLyS and APRIL, effectively suppressing the development and survival of plasma cells and mature B cells (11, 19). Its efficacy and safety have been demonstrated in diseases like systemic lupus erythematosus (SLE) and rheumatoid arthritis (RA), with ongoing studies in various other conditions (11, 20–24). A single-arm, open-label study reported better effectiveness of telitacicept after PE in patients with neuromyelitis optica spectrum disorders (NMOSD) (25). In our case, telitacicept was chosen as a follow-up treatment after limited responses to other treatments, showing satisfactory efficacy. Clinical symptoms, INCAT, and I-RODS scales improved, and anti-NF155 antibody titers remained low without rebound. The treatment was conducted with caution, using a 160 mg dose of telitacicept once, with the treatment cycle determined by B-cell subset monitoring.

There are limitations of our case report: 1) A case report may not be representative enough and the use of Telitacicept may not be appropriate for every AN patient. 2) Causal inference could not be drawn for the efficacy of Telitacicept in treating AN patients. More cases could represent stronger evidence. 3) The case report was produced retrospectively and the medical record did not contain all relevant data. 4) We didn’t perform post-treatment electromyography and nerve conduction velocity for comparison. The data on the total Ig level were insufficient to present. Thus, the findings from our case reports cannot be generalized but could provide a novel idea for treating refractory AN patients.

In conclusion, this is the first case to report treating an NF155+ AN patient with telitacicept and showing a potential therapeutic efficacy. NF155+ AN requires more in-depth research and understanding, and the development of tailored treatments is crucial to enhance patients’ quality of life and neurological functions. Telitacicept might offer a new therapeutic avenue, especially for those with high pathogenic antibody titers or poor response to existing treatments. However, further studies on the safety, recurrence reduction effect, dosage, and treatment cycle of telitacicept in treating NF155+ AN are essential.

## Data availability statement

The original contributions presented in the study are included in the article/supplementary material, further inquiries can be directed to the corresponding author/s.

## Ethics statement

Written informed consent was obtained from the minor(s)’ legal guardian/next of kin for the publication of any potentially identifiable images or data included in this article.

## Author contributions

YR: Writing – original draft. SC: Writing – review & editing. HY: Writing – review & editing.
